# Sterilization of the Unfit

**Published:** 1912-04

**Authors:** G. Henri Bogart

**Affiliations:** Paris, Ill.


For Texas Medical Journal.

### Sterilization of the Unfit.

BY DR. G. HENRI BOGART, PARIS, ILL.

---

There were four young men, boys, indeed, hanged in Chicago one Friday morning in February. There was a wave of sentimental protest went up against the execution, and a strong effort is being made to alter the laws of Illinois so as to annul the death penalty. One of the Chicago daily papers editorially says of this execution:

"The case of the four slayers of Fred Guelzow, two of whom were little more than boys, presented phases that were quite worthy of the most careful consideration by those who had the authority to commute the sentences of death to imprisonment for life.

"One of the boys was a member of a family of twelve children that lived in a house with two rooms. His surroundings had been of the worst, and it seemed almost inevitable from the day of his birth that the fate, such as he met, awaited him.

"Similar conditions surrounded the childhood and youth of the other members of this murderous gang.

"The sad and pitiful circumstance of the murder of Guelzow is simply another illustration of the baleful influence of evil environments—an illustration that is in evidence in every community.

"Could the amount of money that is spent annually in the capture, trial and punishment of criminals be applied in providing preventives by improving the condition of those whose surroundings almost inevitably lead to crime, murders and executions would be reduced to a minimum."

Herein is certainly food for serious thought. In every department of human endeavor we are recognizing the ounce of prevention, and nowhere so much as in medical circles.

Prophylaxis is more considered than cure.

The sane sanitation which has converted the Panama zone from the plague spot of the New World into an ideal health resort is probably the most brilliant example, and the Spanish-American war with its wonderful lessons of heroism and unselfish scientific devotion by surgeons who laid the curb of prevention upon the scourge of yellow fever is one of the epochs of the world's history.

The prevention of such crimes as led to and compelled the other crime of hanging is found in intelligent selection of human birth. "If thine eye offend thee, pluck it out," says theology, and if a breed of evil blood become pronounced in the communal life, it must be eradicated.

The wonderful process of sterilization of the unfit, which prevents these evil births and at the same time preserves the liberties and happiness of the present individuals, is one of the greatest as well as one of the simplest boons that science has conferred upon the process of the elevation of the race.

The making possible of this boon must begin with the physician and continue through the legislative chamber until completed in legal enactment. The most perfect law so far enacted is that which went into force in Iowa July 4, 1911:

"IOWA LAW: CHAPTER 129.

*"An act to prevent the procreation of habitual criminals, idiots, feeble-minded, and imbeciles.* [*Additional to title twelve (XII) of the code relating to the police of the State.*]

"SECTION 1. That it shall be the duty of the managing officer of each public institution in the State, entrusted with the custody or care of criminals, idiots, feeble-minded, imbeciles, drunkards, drug-fiends, epileptics and syphilitics, and they are hereby authorized and directed to annually, or oftener, examine into the mental or physical condition of the inmates of such institutions, with a view of determining whether it is improper or inadvisable to allow any of such inmates to procreate; and to annually, or oftener, call into consultation the members of the State Board of Parole. The members of such board and the managing officer and the surgical superintendent of such institution shall judge of such matters. If a majority of them decide that procreation by any of such inmates would produce children with a tendency to disease, crime, insanity, feeble-mindedness, idiocy or imbecility, and there is no probability that the condition of any such inmate so examined will improve to such an extent as to render procreation by any such inmate advisable, or if the physical or mental condition of any such inmate will be materially improved thereby, or if such inmate is an epileptic or syphilitic, or gives continued evidence while an inmate of such institution that he or she is a moral or sexual pervert, then the surgeon of the institution shall perform the operation of vasectomy or ligation of the Fallopian tubes, as the case may be, upon such person. Provided, that such operation shall be performed upon any convict or inmate of such institution who has been convicted of prostitution or violation of the law, as laid down in chapter two hundred sixteen (216), acts of the Thirty-third General Assembly, or who has been twice convicted of some other sexual offense, or has been three times convicted of felony, and each of such convict or inmate shall be subjected to this same operation of vasectomy or ligation of the Fallopian tubes, as the case may be, by the surgeons of the institution.

"SEC. 2. Except as authorized in this act, every person who shall perform, encourage, assist in or otherwise promote the performance of either of the operations described in Section 1 of this act, for the purpose of destroying the power to procreate the human species, or any person who shall knowingly permit either of such operations to be performed upon such persons, unless the same shall be a medical necessity, shall be fined not more than one thousand ($1000) dollars, or imprisoned in the county jail not to exceed one year, or both."

It will be noted that this law includes all institutions; an improvement upon the Connecticut law; that it is madatory on all; an improvement upon the Indiana law; and that it applies equally to the sexes. The law is automatic on the habitual criminal and sexual pervert, and while the proper use of the operation as a "medical necessity" is allowed, it is restricted as to the promiscuous sterilization of those who would wish the operation as a pretext for unlicensed lust.

This law leaves the enforcement of its provisions to the judgment of trained professional specialists, and not to the whim of laymen, as does the Indiana statute.

On the other hand a law may be so drawn as to destroy its usefulness. Of this type is the California law.

CALIFORNIA LAW.

"Whenever in the opinion of the medical superintendent of any State Hospital, or the superintendent of the California Home for the Care and Training of Feeble-minded Children, or of the resident physician in any State prison, it would be beneficial and conducive to the benefit of the physical, mental, or moral condition of any inmate of said State Hospital, home or State prison, to be asexualized, then such superintendent or resident physician shall call in consultation the general superintendent of State hospitals and the secretary of the State Board of Health, and they shall jointly examine into all the particulars of the case with the said superintendent or resident physician, and if in their opinion, or in the opinion of any two of them, asexualization will be beneficial to such inmate, patient or convict, they may perform the same; provided, that in the case of an inmate or convict confined in any of the State prisons of this State, such operation shall not be performed unless the said inmate or convict has been committed to a State prison in this or some other State or country at least two times for some sexual offense, or at least three times for any other crime, and shall have given evidence while an inmate in a State prison in this State that he is a moral or sexual pervert; and provided, further, that in the case of convicts sentenced to State prison for life who exhibit continued evidence of moral and sexual depravity, the right to asexualize them, as provided in this act, shall apply, whether they have been inmates of a State prison either in this or any other State or country more than one time. (Statutes 1909, p. 1093.)"

It will be observed that the operation of this law is practically confined to the habitual criminal whose procreative course is usually run before he becomes eligible to treatment. The habitual criminal is already provided for in some States in which the third conviction becomes life imprisonment.

The futility of this law as a preventive of the birth of the unfit is so apparent that there is no need more than that I call attention thereto.

Even more absurdly futile is the New Jersey law, which while confined to the same habitual criminal class provides that when recommended for operation the subject *shall be tried by a jury in court.* At that the question will go to the Supreme Court.

The Trenton, New Jersey, *News* says:

"Because of the uncertainty of the constitutionality of the sterilization law, and in order to be free from censure, the State Sterilization Board has decided not to put in operation the provisions of that law until the Supreme Court has been able to pass upon its constitutionality.

"Judge Gnichtel, of the Common Pleas Court, and Assistant Attorney General Nelson B. Gaskill were consulted, and a plan of action was decided upon. One of the cases is to be taken in the regular way before the Mercer County Court of Common Pleas, and the counsel, to be appointed by the court, is to contest, and in that way the matter will be carried before the Court of Errors, so the constitutionality of the act may be passed upon."

The legal phases of the matter have been well handled by the California courts, and this decision will be the theme of a subsequent paper.

---

Texas led the Union last year in the erection of schoolhouses, averaging two daily at a total cost of over $3,000,000. Most of the houses were built without due regard to lighting, heating or ventilating, thus endangering the health of the children and affecting the general efficiency of the schools. It is the duty of school officials and others who are interested in the children of Texas to see that all school buildings erected conform to recognized standards of school architecture. The University of Texas, the Conference for Education in Texas, and the National Bureau of Education, Washington, D. C., will send free on request bulletins on schoolhouses and plans for model buildings, thus making it possible for any community in Texas to have the services of skilled architects.

